# The Effectiveness and Tolerability of Atropine Eye Drops for Myopia Control in Non-Asian Regions

**DOI:** 10.3390/jcm12062314

**Published:** 2023-03-16

**Authors:** Dovile Simonaviciute, Andrzej Grzybowski, Carla Lanca, Chi Pui Pang, Arvydas Gelzinis, Reda Zemaitiene

**Affiliations:** 1 Department of Ophthalmology, Medical Academy, Lithuanian University of Health Sciences, 44037 Kaunas, Lithuania; 2 Department of Ophthalmology, University of Warmia and Mazury, 10-724 Olsztyn, Poland; 3 Institute for Research in Ophthalmology, 60-554 Poznan, Poland; 4 Escola Superior de Tecnologia da Saúde de Lisboa (ESTeSL), Instituto Politécnico de Lisboa, 1549-020 Lisboa, Portugal; 5 Comprehensive Health Research Center (CHRC), Escola Nacional de Saúde Pública, Universidade Nova de Lisboa, 1099-085 Lisboa, Portugal; 6 Department of Ophthalmology and Visual Sciences, The Chinese University of Hong Kong, Hong Kong, China; 7 Joint Shantou International Eye Center of Shantou University, The Chinese University of Hong Kong, Shantou 515051, China; 8 Hong Kong Hub of Paediatric Excellence, The Chinese University of Hong Kong, Hong Kong, China

**Keywords:** myopia progression, myopia treatment, myopia control, atropine eye drops

## Abstract

Myopia is the most common ocular disorder worldwide with an increasing prevalence over the past few decades. It is a refractive error associated with excessive growth of the eyeball. Individuals with myopia, especially high myopia, are prone to develop sight-threatening complications. Currently, atropine is the only drug that is used to slow myopia progression in clinical practice. However, there are still areas of uncertainty such as treatment strategy, optimal concentration when considering risk–benefit ratio and active treatment period. Since the prevalence of myopia is much higher in Asian countries, most of the research on myopia control has been conducted in Asia. Data on the efficacy and tolerability to atropine eye drops in the non-Asian population remains limited. In this review, we summarize the results of published clinical trials on the effectiveness and tolerability of atropine eye drops for myopia control in non-Asian regions. The efficacy was evaluated by the mean change in spherical equivalent (SE) or axial length (AL). The tolerability of atropine eye drops was analyzed based on patients complains and adverse events. The results of this review suggest that 0.01% atropine eye drops are effective in non-Asian regions achieving less side effects compared to 0.5% concentration.

## 1. Introduction

Myopia is the most common ocular disorder worldwide with an increasing prevalence over the past few decades. It is predicted that approximately half of the world’s population will be myopic by 2050 and 10% will have high myopia [1]. The prevalence of myopia among Europeans is up to 30–40%, and it is almost 50% in the 25–29 years of age group [2]. The prevalence of myopia is much higher in Asian countries and currently about 90% of young adults have myopia [3].

Myopia is a refractive error associated with excessive growth of the eyeball. Individuals with myopia, especially high myopia, i.e., spherical equivalent (SE) ≤ −6.0 D or axial length (AL) > 26 mm, are prone to develop sight-threatening complications, such as retinal detachment, myopic choroidal neovascularization, myopic macular degeneration, foveoschisis, glaucoma and cataract [4,5]. Despite the relatively low prevalence of pathologic myopia, it is still a major cause of blindness and visual impairment in both Asian and Western populations. Estimated prevalence of irreversible vision impairment due to pathologic myopia is 1 to 5 per 1000 persons in the European population [6]. Reducing the rate of myopia progression by 50% could reduce the prevalence of high myopia by up to 90% [7]. Since high myopia is associated with sight-threatening conditions, delaying myopia onset and slowing myopia progression in school-aged children is essential for reducing the risk of progression to high myopia and its associated complications later in life [8,9].

Different treatment strategies have been used to prevent myopia development and to reduce its progression. At least 2 hours a day or 14 hours per week outdoors and a reduction in long and intensive near work are recommended to prevent myopia development in children [10]. Interventions to slow myopia progression include optical methods such as bifocal, progressive or defocus-incorporated multiple segments’ spectacles, orthokeratology, contact lenses and atropine eye drops [11]. Currently, atropine is the only drug that is used to slow myopia progression in clinical practice [12]. The most common regimen of atropine usage is one drop of 0.01–0.05% atropine in the evening in both eyes for at least one or two years or even longer in children from 5 to 15 years of age with myopia progression [13]. Though treatment with low-concentration atropine eye drops is promising in myopia control, there are still areas of uncertainty such as treatment strategy, optimal concentration when considering the risk–benefit ratio, active treatment period and why does the response to the treatment vary among the spectrum of treated myopic children.

Low-dose atropine has been shown to be effective in slowing myopia progression in children in large randomized clinical trials in Asia [14,15]. Since the prevalence of myopia is much higher in Asian countries, most of the research on myopia control have been conducted in Asia. Data on efficacy and response to atropine eye drops in non-Asian populations remain limited. Based on studies performed in Asia, 0.05% atropine eye drops have been recommended as the optimal dosage considering the risk–benefit ratio [14]. However, impaired vision or reading difficulties were found in 63.0% of Caucasian children treated with 0.05% atropine eye drops [16]. Studies show a higher incidence and more pronounced side effects of 0.05% atropine eye drops in Caucasian children than those observed in Asian populations [16,17]. The optimal concentration for the treatment of myopia progression in non-Asian populations remains unclear. Therefore, the aim of this review is to describe the results of clinical trials on atropine eye drops for myopia progression in non-Asian regions.

## 2. Materials and Methods

In this review, we searched the PubMed and Web of Science databases for studies conducted up to 10 September 2022. The following keywords were used: myopia progression treatment, myopia progression control, atropine eye drops. Randomized controlled trials (RCTs), prospective and retrospective studies were included if they analyzed atropine eye drops’ usage for myopia progression in non-Asian regions with a treatment duration of at least 1 year. Articles published from 2012 to 10 September 2022 were included. We extracted the following information from each trial: follow-up duration, sample size and ethnicity, age, mean change in refraction and axial length and information on side effects and adverse events. The inclusion criteria were as follows: (1) studies on atropine usage for myopia control published up to 10 September 2022; (2) children with myopia aged <18 years; (3) follow-up period of 1 year or more; (4) studies performed in non-Asian regions with most of the enrolled subjects of non-Asian ethnicity; (5) studies reporting at least 1 outcome of interest, including the annual rate of myopia progression (changes of SE under cycloplegia or AL); (6) studies written in the English language. Studies were excluded if they (1) did not evaluate changes in AL and SE was evaluated without cycloplegia, (2) analyzed a combination of atropine and other means of myopia control or (3) were review papers.

## 3. Results

**Figure 1:**
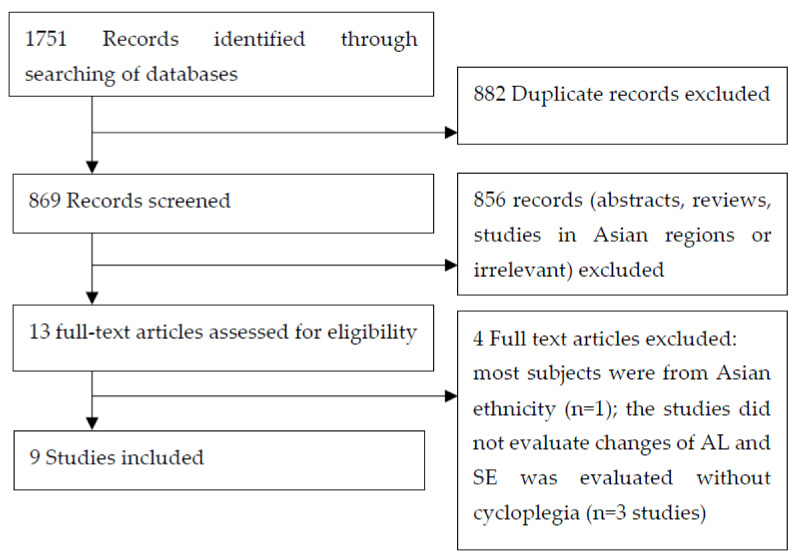
Flowchart of study selection process.

A total of nine studies (three RCTs, four prospective and two retrospective studies) met the study criteria and were included in this review (Figure 1). The summary of the studies is presented in Table 1, Table 2 and Table 3.

### 3.1. 0.5% Atropine Eye Drop

#### Measurements of SE with Cycloplegia

A study performed in the Netherlands showed that 0.5% atropine eye drops were effective in slowing myopia progression in children [20]. The mean SE progression rate before treatment was −1.0 D (SD 0.7) a year and after 1 year with treatment decreased to -0.1 D (SD 0.7) a year. Most of the subjects were European people (68.8%), but 23.4% of Asian people were included in the study. However, further detailed analysis of the results showed that ethnicity did not have influence on the 0.5% atropine eye drops efficacy. A relatively high frequency of adverse events was observed. Almost 83% of the treated children complained about side effects of atropine eye drops. The most prominent were photophobia (72%), followed by reading problems (38%) and headaches (22%) (Table 4).

Pooling and colleagues performed a 3-year follow-up study with children treated with 0.5% atropine eye drops for progressive myopia [21]. Most children (66.9%) were European. One third of treated children (36%) had an insufficient response to treatment (SE ≥ −1 D/year and AL increased ≥0.3 mm/year) and after 1 year 1.0% atropine was prescribed. Despite that, the progression rate remained similar. Twenty-nine percent showed a good response to treatment (SE < −0.5 D/year and AL < 0.2 mm/year) with 0.5% and the atropine concentration was reduced to 0.25% and further to 0.1% and 0.01% every 6 months if myopia remained stable. Thirty five percent of patients remained on the treatment with 0.5% concentration during the study follow-up. During the 3-year follow-up, the median annual progression of SE and AL for treated children was −0.25 D and 0.11 mm, respectively (median SE refraction and AL change were 0.00 D in the 1st year, −0.41 D and −0.38 D in the second and third year and 0.04 mm in the first year and 0.16 mm and 0.14 mm in the second and third year, respectively). Changes in AL were only evaluated during the treatment period. Fourteen percent of children discontinued atropine eye drops due to adverse events and 7% because of allergy. Both studies performed in the Netherlands [20,21] did not provide a control group and SE progression was assessed one year before the beginning of the treatment and during the treatment in the same subjects. Studies with 0.5% atropine eye drops had dropout rates of about 22%.

### 3.2. 0.01% Atropine Eye Drops

#### 3.2.1. Measurements of SE with Cycloplegia

A 2-year RCT study in Spain revealed that 0.01% atropine eye drops were effective in slowing the progression of myopia based on the changes in both SE and AL [23]. At the 2 years follow-up visit, SE changed by −0.51 (SD 0.39) D in the atropine treatment group vs. −0.76 (SD 0.37) D in the control group, (*p* < 0.001). AL increased by 0.20 (SD 0.20) mm vs. 0.37 (SD 0.27) mm, (*p* < 0.001), respectively. Only 1 patient from 171 patients was excluded from the study because of mydriasis and blurred near vision after usage of atropine eye drops.

The results of a 5-year RCT study in Spain concluded that annual myopia progression rate was −0.14 (SD 0.35) D in the 0.01% atropine treatment group against −0.65 (SD 0.54) D in the control group [22]. A statistically significant difference was observed in SE between the groups after usage of 0.01% atropine eye drops but only after two years of treatment. Side effects (such as photophobia, reading difficulty, mydriasis, and headache) that required withdrawal of the treatment occurred in only 2% of the treatment group. The study showed that 0.01% atropine eye drops do not have serious side effects even after long-term use. Eighteen children discontinued atropine eye drops after 2 years of treatment and the progression of myopia was −0.43 (SD 0.36) D per year during the 3 years follow-up, showing that myopia progression increased by up to three-fold after discontinuation of the treatment.

Perez-Flores et al. conducted a multicenter study that also proved 0.01% atropine eye drops have efficacy and safety in a Spanish cohort [18]. Mean SE progression before treatment was −1.01 (SD 0.38) D a year versus −0.44 (SD 0.41) D a year while using 0.01% atropine eye drops (*p* < 0.001). Mean AL change was 0.27 (SD 20) mm after one year of treatment. The study did not have a control group and did not evaluate the change of AL before treatment. After one year of treatment, 1.1% (one patient) of children complained of mild ocular discomfort, 2.2% (two patients) had mild light intolerance and 2.2% (two patients) had near vision difficulties. At the 2 weeks follow-up, 15.2% (14 patients) complained of mild ocular discomfort, 7.6% (7 patients) experienced photophobia and 5.4% (5 patients) had near vision difficulties. However, all the side effects were mostly mild and transitory and only four patients withdrew from the study due to adverse reactions.

A retrospective analysis of 13 myopic Australian children aged from 2 to 18 years old treated with 0.01% atropine eye drops also confirmed its effectiveness [19]. According to the progression of myopia before treatment, children were classified as “slow” (a mean rate of SE of −0.19 (SD 14) D a year) and “fast” (a mean rate of SE progression of −1.01 (SD 0.56) D a year) myopia progressors. During the treatment, SE decreased by −0.07 D a year in the “slow” progression group and -0.25 D a year in the “fast” progression group (*p* = 0.03). The study did not have a control group and the authors compared AL data to the age-matched data from the published literature. In the “slow” progressors group, atropine eye drops slowed AL compared to the age-matched data of untreated myopes (0.098 mm/year compared to 0.20 mm/year, *p* < 0.001), but did not have any effect on AL in the “fast” progressors group (0.27 mm/year and 0.25 mm/year, respectively, *p* = 0.754). Minor side effects, such as pupillary dilatation, were observed in six cases and were more common in children with a blue iris. Dry or irritated eyes were observed in five children. However, because of the small sample size, no significant association was found between iris color and adverse reactions.

Sacchi and colleagues analyzed medical records of 52 patients treated with 0.01% atropine eye drops and 50 control subjects [25]. After 12 months, the mean myopia progression rate was −0.54 (SD 0.61) D in the 0.01% atropine eye drops group and −1.09 (SD 0.64) D (*p* < 0.001) in the control group. However, 21% of treated patients (11 children) showed a progression of >0.50 D despite the treatment. Only five patients complained of temporary photophobia.

A RCT study by Lee et al. showed that 0.01% atropine eye drops had a modest myopia control effect in multi-racial Australian children [24]. Statistically significant differences in SE and AL between the 0.01% atropine eye drops group and the placebo group were found at 6, 12 and 18 months, but not at 24 months. The study included 153 children (104 received 0.01% atropine eye drops and 49 received placebo eye drops). After one year, mean SE and AL change from baseline were −0.31 D (95% confidence interval (CI) = −0.39 to −0.22) and 0.16 mm (95% CI = 0.13–0.20) in the atropine group vs. −0.53 D (95% CI = −0.66 to −0.40) and 0.25 mm (95% CI = 0.20–0.30) in the placebo group (*p* < 0.01). However, after two years the difference between the groups was not statistically significant. Mean SE and AL change from baseline was −0.64 D (95%CI = −0.73 to −0.56) and 0.34 mm (95%CI = 0.30–0.37) in the atropine group, and −0.78 D (95%CI = −0.91 to −0.65) and 0.38 mm (95%CI = 0.33–0.43) in the placebo group (*p* = 0.10). These results could be related with the older age in the placebo group (one-year), due to children with fast progressive myopia who were allocated to the placebo group who withdrew from the study and sought alternative treatment. Most of the included children were European, but there were 40.8% and 38.4% of Asian children in the placebo and the 0.01% atropine eye drops groups, respectively. There was no statistically significant difference in change in SE or AL between the placebo and atropine groups in Asian children over the 2 years period. There was no statistically significant difference in the incidence of adverse events between the atropine and control groups (*p* = 0.17), but children in the atropine group had reduced accommodative amplitude and pupillary light response compared to the placebo group.

The results of Phase III CHAMP (Childhood Atropine for Myopia Progression) clinical study on low dose atropine (0.01%) including a large USA and European population have been recently presented and seemed to confirm the effectiveness of 0.01% in non-Asian children [27]. Children on nightly dosing with 0.01 atropine eye drops were followed-up for 3 years and compared with a placebo group. Adverse events were lower when compared with 0.02% with no treatment discontinuation. The main results of this study have not been published yet and may provide further insights into myopia treatment with 0.01% atropine eye drops in non-Asian children.

#### 3.2.2. Measurements of SE without Cycloplegia

Most of the included studies evaluated SE under cycloplegia (Table 5). However, the study by Kaymak et al. performed cycloplegia only in some cases and evaluated changes of AL. A one-year retrospective analysis of 0.01% atropine eye drops on myopia progression in a routine clinical setting showed an inhibition of 0.08 mm per year for AL growth in the atropine group in a German population [26]. Fifty-one percent of subjects in the atropine group had progression of AL less than 0.2 mm/year, 26% progressed by 0.2 to 0.35 mm/year and 23% progressed by more than 0.35 mm/year. In the control group, the proportions were 47%, 28% and 25%, respectively (*p* < 0.0015). However, the effects on refraction were not statistically significant between the groups. The reasons for these equivocal results may be because cycloplegia was not performed in all the cases and atropine eye drops were not prescribed every evening, but only 5 days per week. Additionally, neither the baseline refraction nor the AL or age were similar between the groups. None of the children had serious complications, but 16.7% of the atropine-treated children reported side effects such as burning eyes after drops’ instillation, pupil dilatation, photophobia or eye redness.

## 4. Discussion

In this review, we analyzed the efficacy and side effects of 0.01% and 0.5% atropine eye drops for the treatment of myopia progression in non-Asian regions. The results of this review suggest that 0.01% atropine eye drops are effective in controlling myopia progression in non-Asian regions, leading to less side effects compared to 0.5% atropine eye drops. Most of the included studies showed the effectiveness of 0.01% atropine eye drops in slowing the progression of myopia. Nevertheless, some study subjects did not respond to treatment or treatment effect was limited to 1.5 years. About 20% of children were non-responders in the study by Moriche-Carretero et al. (SE increase of >1 D/2 years) [23], and 36% in the study by Polling et al. [21]. In the latter report, an atropine eye drops’ dose as high as 1% did not diminish myopia progression. The study by Lee and colleagues was the only study that included a placebo group and showed 0.01% atropine eye drops effectiveness after 1.5 years, but did not after 2 years [24]. In addition, the study by Lee et al. also analyzed the effectiveness of 0.01% atropine eye drops separately for Asians and Caucasian people and did not find a statistically significant difference compared to the control groups of Asian and Caucasian people after 2 years [24]. The study by Polling et al. showed that 0.5% atropine eye drops are equally effective in both Caucasian and Asian people [20].

Both studies by Polling and colleagues [20,21] analyzed the efficacy of 0.5% atropine eye drops and showed a higher incidence of adverse events compared to studies with 0.01% atropine eye drops. These studies with 0.5% atropine eye drops had more dropouts (around 22%) than those reported in Asian studies (around 14.0%) with the same concentration atropine eye drops [28]. It is hypothesized that the pupil-dilating effect of atropine eye drops is less common in Asians due to their more pigmented irises. A one year study with 0.5% atropine eye drops concluded that 83% of treated patients experienced adverse events [20]. Joachimsen et al. analyzed the differences of side effects between 0.01% and 0.05% atropine eye drops in myopic German schoolchildren [16]. They found that children treated with 0.05% atropine eye drops showed significantly higher anisocoria and loss of accommodation amplitude (AA) compared to children treated with 0.01%. Side effects were more pronounced in Caucasians treated with 0.05% atropine eye drops compared to reported side effects in Asian children. In the Low-Concentration Atropine for Myopia Progression (LAMP) study [17], photopic pupil size increased by 1.1 mm, while in the German study it increased by 2.9 mm in the 0.05% atropine treatment group, and AA decreased by 2.4 D and 4.2 D, respectively (Table 6). Sixty-three percent of Caucasian children treated with 0.05% atropine eye drops experienced a decrease in near visual acuity or reading difficulties. The 0.01% atropine eye drops had minimal side effects on pupil size, accommodation and near vision.

In a Spanish cohort a mean increase of 0.74 mm in pupil diameter (PD) was observed when using 0.01% atropine eye drops [18]. Cyphers and colleagues evaluated symptoms and ocular findings associated with the use of 0.01% atropine eye drops for one week in young adults aged 21–30 years [29]. Thirty-one participants, of whom 81% were Caucasian, did not report any side effects. An increase in photopic pupil size by 0.2 mm and average intraocular pressure of the two eyes by 1.1 mmHg were found, but none of the changes were clinically meaningful. Neither vision, nor accommodation or reading speed were affected by the use of 0.01% atropine eye drops for one week. A meta-analysis by Huy and colleagues analyzed the effect of various concentrations of atropine eye drops on PD and AA [30]. Only 3 of the 13 studies provided data on PD and 4 of the 13 on AA. All these studies were conducted in Asian countries. The meta-analysis results showed that all the analyzed concentrations (0.01%, 0.02%, 0.025%, 0.05%, 0.1%, 0.5% and 1.0%) resulted in a reduction in AA (mean change −4.67 D, 95% CI, −7.44 to −1.89D, *p* < 0.001) and an increase in PD (mean increase 1.33 mm, 95% CI, 0.57 to 2.09 mm, *p* < 0.001) except for 0.01% atropine. In concentrations lower than 0.1%, the slope of the curve between atropine and change in PD and AA, was steep but changes were smaller, whereas at concentrations higher than 0.01% the slope plateaued with higher changes in AA and PD. Another meta-analysis by Ha and colleagues compared the safety of eight different concentrations of atropine [31]. Most of the included studies were performed in Asian regions. The results showed that atropine has a higher mean difference (MD) of photopic and mesopic PD relative to the control group (concentration from 0.01–0.5%), ranging from MD of 0.59 mm (95% CI, 0.16–1.01 mm for 0.01%) to 2.96 mm (95% CI, 2.00–3.91 mm for 0.5% atropine) and MD ranging from 0.13 mm (95% CI, −0.02–0.28 mm for 0.01%) to 2.54 mm (95% CI, 2.20–2.88 mm for 0.5% atropine), respectively. Among six different concentrations (0.01%, 0.02%, 0.025%, 0.05%, 0.1%, 0.5%) 0.5% (MD, −7.65; 95% CI, −10.44 to −4.85) and 0.01% (MD, −5.95; 95% CI, −8.73 to −3.16) atropine eye drops showed a lower MD for AA relative to the control group. From five different concentrations (0.01%, 0.025%, 0.05%, 0.1%, 0.5%) only 0.1% atropine had an effect on distance best-corrected visual acuity. The study by Loughman and Flitcroft analyzed the impact of 0.01% atropine on visual performance and quality of life in 14 Caucasians aged 18–27 years [32]. The effect of atropine eye drops was statistically significant for pupil size and responsiveness. Reduction in AA was observed, although the change was not significant. Neither visual acuity (near and far) nor reading speed was affected. There was a slight increase in complaints such as glare, but overall, there was no impact on quality of life. Thus, although the reported efficacy in controlling myopia progression of 0.01% atropine eye drops is lower than that of higher doses of atropine in Asian populations [14,17], it is widely accepted in Europe due to its minimal side effects. 

The rebound effect, when myopia progresses faster than usual after discontinuation of atropine eye drops, has been assessed in several studies. Diaz-Llopis et al. [22] found that myopia progressed up to three times faster after withdrawal of the treatment. In a retrospective analysis by Myles et al., two patients who discontinued atropine eye drops experienced myopia progression of −1.12 D/year in three out of four eyes compared to the progression of −0.51 D/year during treatment [19].

Results of this study revealed that there is a lack of prospective, randomized-controlled trials in non-Asian populations that analyzed the effect of atropine eye drops on the myopia progression. From the nine included studies, only three were RCTs. This review has several limitations that should be highlighted. Children of mixed ethnicities were included in the studies performed in the USA, Australia and some European countries. Adherence and compliance to the treatment regimen was not accurately assessed in most of the studies. The effectiveness and tolerability were analyzed only using 0.01% or 0.5% atropine eye drops, and there is a lack of studies with other concentrations in non–Asian regions. Additionally, not all the studies had a control group. In addition, there was a considerable variation in the age of children and the degree of myopia. In some studies, children with myopia were included regardless of the rate of progression and others defined SE or determined myopia progression by > 0.5 D or >1.0 D over a year. Considering that progression of myopia slows down with age, this may have influenced the results.

## 5. Conclusions

In this review we analyzed the efficacy and side effects of 0.01% and 0.5% atropine eye drops for the treatment of myopia progression that were reported in non-Asian countries. Recent studies in Asian countries suggest 0.05% atropine eye drops as the optimal concentration. The results of this review suggest that 0.01% atropine eye drops are effective in non-Asian children achieving less side effects compared to 0.5% atropine eye drops Studies with longer follow-up and with different atropine concentrations are necessary, as well as more studies being needed that evaluate AL changes or the rebound effect to provide evidence-based information on the effect and adverse events of atropine eye drops on myopia progression in non-Asian children.

## Figures and Tables

**Table 1:** Characteristics of prospective studies included in the analysis.

Author, Year	Country	Number of Children and Ethnicity	Study Design	Age (y)	Mean Baseline SE (D); Inclusion SE (D); Astigmatism (D)	Follow -Up Time (Months)	Change of SE (D) Control Group	Change of SE (D) Atropine Group	Mean Baseline AL (mm)	Change of AL (mm) Control Group	Change of AL (mm) Atropine Group
Pérez-Flores et al., 2021 [18]	Spain	n = 92: 0.01% Atropine group Caucasian (n = 90) Asian (n = 2)	Prospective study	6–14	SE N/A; −2.00 to −6.00 and progression ≥ 0.5 D/year; <1.50	0–12	−1.1 (Progression of the previous year)	−0.44	24.57	N/A	0.27
Myles et al., 2021 [19]	Australia	n = 13: 0.01% Atropine group (n = 13)	Prospective study	2–18	−0.25; N/A	0–60	“slow” progressors -0.19 D “fast” progressors -1.01 D (Progression of the previous year)	“slow” progressors −0.07 D “fast” progressors -0.25 D	N/A	“Slow” progressors 0.196 mm/year “fast” progressors 0.245 mm/year (published data of untreated myopes)	“slow” progressors 0.098 mm/year “fast progressors” 0.265 mm/year
Polling et al., 2016 [20]	Netherlands	n = 77: 0.5% Atropine group European (n = 53) Asian (n = 18) African (n = 6)	Prospective	3–17	SE −6.6; ≤−3.0 and progression ≥ 1.0 D/year; N/A	0–12	−1.0 (over 12 months before treatment)	0.1	25.19	N/A	0.35
Polling et al., 2020 [21]	Netherlands	n = 124: 0.5% Atropine group European (n = 83) East Asian (n = 13) Other (n = 29)	Prospective	5–16	SE −5.03; Progression >1.0 D/year or SE ≤ −2.5 in children ≤10 or SE ≤ −5.0 in children ≥ 11 years. N/A	0–12 12–24 24–36	−1.1 (over 12 months before treatment)	+0.1 −0.3 −0.3	25.14	N/A	0.0 0.1 0.1

y: year; D: diopter; SE: spherical equivalent; AL: axial length; N/A: not applicable.

**Table 2:** Characteristics of RCTs included in the analysis.

Author, Year	Country	Number of Children and Ethnicity	Study Design	Age (y)	Mean Baseline SE (D); Inclusion SE (D); Astigmatism (D)	Follow -Up Time (Months)	Change of SE (D) Control Group	Change of SE (D) Atropine Group	Mean Baseline AL (mm)	Change of AL (mm) Control Group	Change of AL (mm) Atropine Group
Diaz-Llopis et al., 2018 [22]	Spain	n = 200: 0.01% Atropine group Caucasian (n = 100) Control group Caucasian (n = 100)	Randomized Controlled Trial	9–12	SE control group −1.2, Atropine group −1.1; −0.5 to −2.00; <1.5	0–12 0–60	−0.65 −3.25	−0.14 −0.7	N/A	N/A	N/A
Moriche-Carretero et al., 2021 [23]	Spain	n = 339: 0.01% Atropine group Caucasian (n = 171) Control group Caucasian (n = 168)	Randomized Controlled Trial	5–11	SE −2.15; −0.5 to −4.5; ≤ 1.50	0–24	−0.76	−0.51	24.24	0.37	0.20
Lee et al., 2022 [24]	Australia	n = 153: Atropine group European (n = 52) East Asian (n = 18) South Asian (n = 22) Other (n = 12) Placebo group European (n = 23) East Asian (n = 9) South Asian (n = 11) Other (n = 6)	Prospective double-masked, randomized, placebo-controlled study	6–16	SE placebo group −3.56, atropine group −3.13; ≤−1.50 and progression ≥0.5 D/year; ≤1.50 D;	0–6 0–12 0–18 0–24	−0.36 −0.53 −0.74 −0.78	−0.13 −0.31 −0.49 −0.64	Placebo group 24.7 Atropine group 24.6	0.16 0.25 0.35 0.38	0.07 0.16 0.25 0.34

y: year; D: diopter; SE: spherical equivalent; AL: axial length; N/A: not applicable.

**Table 3:** Characteristics of retrospective studies included in the analysis.

Author, Year	Country	Number of Children and Ethnicity	Study Design	Age (y)	Mean Baseline SE (D); Inclusion SE (D); Astigmatism (D)	Follow -Up Time (Months)	Change of SE (D) Control Group	Change of SE (D) Atropine Group	Mean Baseline AL (mm)	Change of AL (mm) Control Group	Change of AL (mm) Atropine Group
Sacchi et al., 2019 [25]	Italy	n = 102: 0.01% Atropine group Caucasian (n = 52) Control group; Caucasian (n = 50)	Retrospective	5–16	SE control group −2.63, atropine group −3.0; SE progression ≥0.5 D/year; N/A	0–12 (without treatment) 12–24 (with atropine eye drops)	−0.80 (control group during 1st year) −1.09 (control group during 2nd year) −1.20 (atropine group 1-year before treatment)	−0.54	N/A	N/A	N/A
Kaymak et al., 2021 [26]	Germany	n = 183: 0.01% Atropine group Caucasian (n = 80) Control group Caucasian (n = 103)	Retrospective study	3–15	SE control group −2.92, atropine group −4.21; −0.125 to −15.25; N/A	0–12	N/A	N/A	AL control group 24.40; Atropine group 24.82	N/A	Inhibition of 0.08 mm (28%) per year

y: year; D: diopter; SE: spherical equivalent; AL: axial length; N/A: not applicable.

**Table 4:** Comparison of frequency of adverse events between studies included in the analysis.

Author, Year	Atropine Concentration	Dropout Rate Because of Side Events	Reported Side Events
Diaz-Llopis et al., 2018 [22]	0.01%	2% (photophobia, reading difficulties, mydriasis and headache)	5% (slight photophobia)
Sacchi et al., 2019 [25]	0.01%	0%	10% (temporary photophobia)
Moriche-Carretero et al., 2021 [23]	0.01%	1% (mydriasis and blurred vision)	-
Pérez-Flores et al., 2021 [18]	0.01%	4% (tachycardia, vertigo and ocular discomfort)	15% at 2 weeks visit (photophobia, blurred near vision)6% at 12 months (mild ocular discomfort, photophobia and blurred near vision)
Kaymak et al., 2021 [26]	0.01%	0%	17% (ocular discomfort, mydriasis, photophobia, redness of the eye)
Myles et al., 2021 [19]	0.01%	23% (eye discomfort, mydriasis, photophobia and headache)	69% (mydriasis, ocular discomfort, photophobia and headache)
Lee et al., 2022 [24]	0.01%	0%	6% (ocular discomfort and blurred near vision)
Polling et al., 2016 [20]	0.5%	22%	83% (photophobia, reading difficulties, headache, systemic flushes, conjunctivitis and others)
Polling et al., 2020 [21]	0.5%	21% (allergic reactions, photophobia and non-eye related causes)	N/A

N/A: not applicable.

**Table 5:** Comparison between the studies included in the analysis.

Author, Year	Cycloplegic Refraction	Control Group	Period of Treatment with Atropine Eye Drops
Diaz-Llopis et al., 2018 [22]	Yes	Yes	2 to 5 years
Sacchi et al., 2019 [25]	Yes	Yes	1 year
Moriche-Carretero et al., 2021 [23]	Yes	Yes	1 year
Pérez-Flores et al., 2021 [18]	Yes	No	1 year
Kaymak et al., 2021 [26]	No	Yes	1 year
Myles et al., 2021 [19]	Yes	No (only data from published studies of untreated myopes)	1 to 5 years
Lee et al., 2022 [24]	Yes	Yes	2 years
Polling et al., 2016 [20]	Yes	No	1 year
Polling et al., 2020 [21]	Yes	No	3 years

**Table 6:** Side effects of 0.01% and 0.05% atropine eye drops.

Characteristic	0.05% Atropine Eye Drops LAMP Study (Asian) [17]	0.05% Atropine Eye Drops German School Children (Caucasian) [16]	0.01% Atropine Eye Drops German School Children (Caucasian) [16]
Change in photopic pupil size	1.1 mm	2.9 ± 1.1 mm	0.8 ± 0.7 mm
Change in accommodation amplitude	−2.4 D	−4.2 ± 3.8 D	−0.05 ± 2.5 D
Complained about visual impairment	19%	63%	N/A
Near vision	0.00 ± 0.13 logMAR	0.05 ± 0.06 logMAR	0.01 ± 0.06 logMAR

N/A: not applicable.

## Data Availability

Data supporting the findings of this study are available within the included articles or published studies.
